# Living Elderhood: A Practical Theology of Old Age According to W. Andrew Achenbaum

**DOI:** 10.1093/geroni/igaf122.1259

**Published:** 2025-12-31

**Authors:** Stephen Fogle

**Affiliations:** At-Large, Omaha, Nebraska, United States

## Abstract

Collaboration between Humanities & Gerontology is poised for a breakthrough. After decades of research operating through Successful and Positive Aging paradigms, gerontologists dedicated to understanding the ‘good stuff’ of old age are charting a complementary course for, “Innovative Horizons in Gerontology.” Inspired by the religion, spirituality, and aging writing of W. Andrew Achenbaum, this paper forwards collaborative exchange between Humanities & Gerontology through presentation of a practical theology of old age called Living Elderhood. Composed in three parts, the Living Elderhood framework focuses attention to 1) recognition of old age; 2) responsibility to love; 3) relationship with Divinity. The paper opens with a brief historical review of Elderhood as a concept of interest across interdisciplinary perspectives from humanities, social sciences, and medicine in gerontological literature. The paper then dives deep into the religion, spirituality, and aging writing of W. Andrew Achenbaum to explicate the dimensions of Living Elderhood expressed through his personal and professional reflections. In doing so, the paper seeks to include religion and spirituality as a part of Achenbaum’s legacy of connecting gerontological and humanities imaginations. The paper concludes with describing how the three dimensions of the Living Elderhood framework are manifested in the lived experiences of older Jesuit priests. Attendees for this paper will 1) become conversant in broader currents of gerontological and humanities collaboration; 2) gain appreciation of W. Andrew Achenbaum as a religious and spiritual thinker; 3) understand application of Living Elderhood in researching older adults’ lived experiences.

